# Periorbital oedema and surgical emphysema, an unusual complication of a dental procedure: a case report

**DOI:** 10.4076/1757-1626-2-8108

**Published:** 2009-09-01

**Authors:** Asif Parkar, Claire Medhurst, Mohammad Irbash, Carl Philpott

**Affiliations:** Accident and Emergency Department, Colchester University Hospital NHS Foundation TrustColchester- CO4 5JLUK

## Abstract

**Introduction:**

We report a case of subcutaneous emphysema and periorbital oedema following a dental procedure.

**Case presentation:**

A 55-year-old female who attended Accident and Emergency department with subcutaneous emphysema of the neck and periorbital oedema several hours after having undergone root canal treatment. She was admitted for prophylactic intravenous antibiotics and was discharged the next day with oral antibiotics and recovered completely in about 10 days.

**Conclusion:**

Although there are existing case reports documenting the occurrence of surgical emphysema following dental procedure, there was no literature documenting a case of periorbital oedema. This can be managed with close observation and antibiotic prophylaxis as in this case but it is important that the potential seriousness of such a complications resulting from dental procedures are not overlooked.

## Introduction

Surgical emphysema is defined as gas or air trapped in the subcutaneous tissue. Common causes giving this condition are tracheotomy, direct laryngoscopy, and oesophagoscopy [1,2]. Subcutaneous and mediastinal emphysema have been previously reported after dental and oral surgical procedures, but remains a rare complication. There appears to be no evidence in the literature to date to link periorbital oedema with dental treatment.

## Case presentation

**Figure 1.**
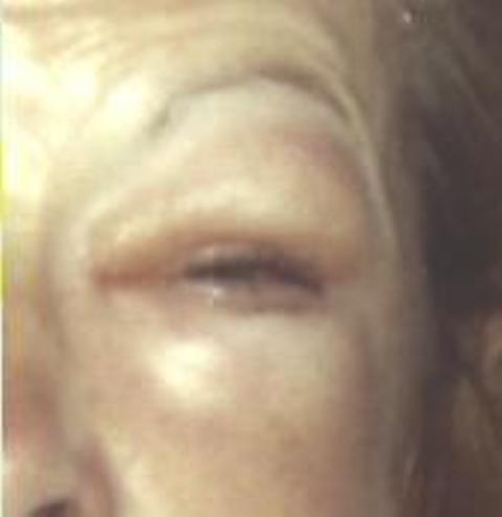
Periorbital oedema and facial swelling on left side.

**Figure 2.**
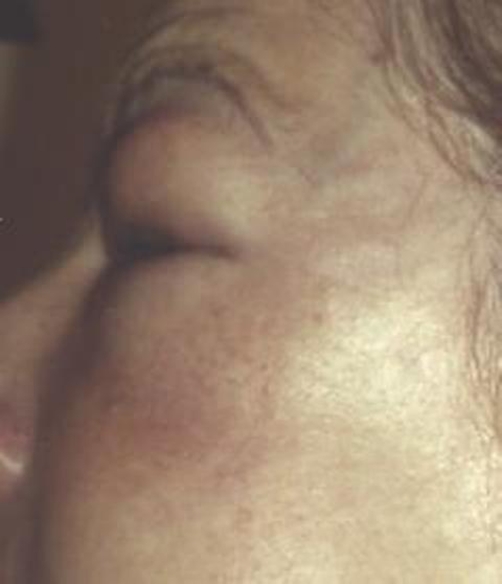
Surgical Emphysema over the face and neck.

**Figure 3.**
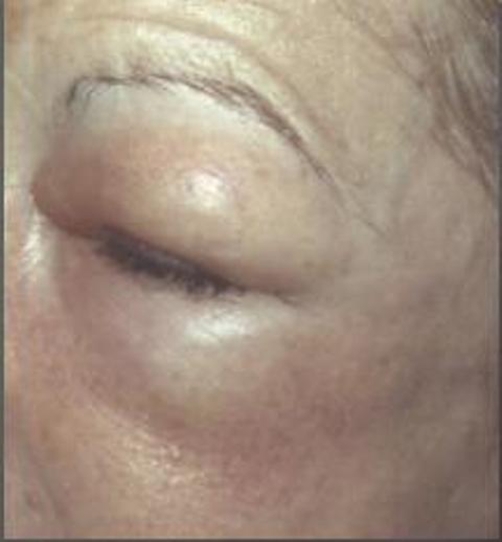
Periorbital oedema.

A 55-year-old British Caucasian female presented to Accident and Emergency department with swelling and discomfort around left eye which developed within an hour following a root canal treatment for left upper molar tooth under local anaesthetic. She was diagnosed as allergic reaction to local anaesthetic, treated with antihistaminic and discharged. She returned to the A & E six hours later with inability to open her left eye due to severe periorbital oedema and surgical emphysema of left side of face and neck with a palpable crepitus (Figures 1-3). She did not complain of respiratory distress, hoarseness, chest pain, dysphagia or odynophagia. She was haemodynamically stable and apyrexial. Plain radiographs of chest, neck and face were done which showed no evidence of pneumothorax but lateral neck radiograph showed air in the prevertebral tissue confirming surgical emphysema of the neck which had no connection with the chest. The patient was given a stat dose of intravenous steroids and antihistamine. She was started on prophylactic intravenous antibiotics and admitted for observation. The patient showed signs of satisfactory recovery and partial opening of her eye after 10 hours. Next day she showed significant recovery with decreased periorbital oedema, surgical emphysema and was discharged home on oral antibiotics. She recovered completely in about 10 days.

## Discussion

Surgical emphysema is a known complication of root canal treatment however periorbital oedema following a dental procedure is what makes this case report interesting. The causes of surgical emphysema include traumatic facial injury, rupture of pulmonary bulla, prolonged surgical procedure, direct injection of air during dental procedure, as in the case described in this report [3]. The first reported case of subcutaneous emphysema following a dental procedure was by Turnball in 1900, who described facial emphysema after premolar extraction [4]. This came after the replacement of earlier, less patient friendly dental hand operated drills with more efficient rotary drills in 1870 and soon after the electric dental drill [5]. The high speed air turbine drills used in dental surgery today are similarly associated with this complication. The pressurised air from the drill is forcefully injected into surrounding subcutaneous tissues in the facial planes. The roots of the first, second and third molars communicate directly with the sublingual and submandibular spaces. The sublingual space is also in direct communication with the pterygomandibular, Para pharyngeal and retropharyngeal spaces. After a dental procedure, the roots may give way to injected air and result in surgical emphysema. However, in this case the oedema around the eye was not typical of surgical emphysema which has a characteristic palpable crepitus. The mechanism for this occurrence is not clear but may result in fluid being forced subcutaneously from the site of the dental treatment in the upper jaw. The normal saline used during the procedure would most likely contain contaminants from the oral cavity, thus there is a risk of infection, which is why our patient was treated with antibiotics.

## Conclusion

Unlike in other reported cases, our patient had none of the rarer, more serious or fatal complications of surgical emphysema following dental treatment such as temporary auditory disturbances, orbital emphysema, retinal artery collapse, optic nerve damage, tension pneumothorax or pneumoperitoneum [1,6-8]. It is not uncommon for patients to present to the Emergency department with dental problems for which accurate advice and specialist treatment may be lacking where local maxillofacial or dental surgical services are not immediately available. These include post extraction bleeding and dental abscesses. We believe this case highlights a rare but important complication of dental procedures that may attend the emergency department which can have serious consequences for the patient if not identified and treated promptly.

## Abbreviation

A & E: Accident and Emergency
